# Randomized clinical trial shows no substantial modulation of empathy-related neural activation by intranasal oxytocin in autism

**DOI:** 10.1038/s41598-021-94407-x

**Published:** 2021-07-23

**Authors:** Annalina V. Mayer, Anne-Kathrin Wermter, Sanna Stroth, Peter Alter, Michael Haberhausen, Thomas Stehr, Frieder M. Paulus, Sören Krach, Inge Kamp-Becker

**Affiliations:** 1 Department of Psychiatry and Psychotherapy, Social Neuroscience Lab, University of Lübeck, Lübeck, Germany; 2 Department of Child and Adolescent Psychiatry, Psychosomatics and Psychotherapy, Philipps University of Marburg, Marburg, Germany; 3 Marburg Center for Mind, Brain and Behavior (CMBB), Philipps University of Marburg, Marburg, Germany; 4 Department of Medicine, Pulmonary and Critical Care Medicine, and Member of the German Center for Lung Research (DZL), Philipps University of Marburg, Marburg, Germany

**Keywords:** Translational research, Empathy, Autism spectrum disorders

## Abstract

Evidence suggests that intranasal application of oxytocin facilitates empathy and modulates its underlying neural processes, which are often impaired in individuals with autism spectrum disorders (ASD). Oxytocin has therefore been considered a promising candidate for the treatment of social difficulties in ASD. However, evidence linking oxytocin treatment to social behavior and brain function in ASD is limited and heterogeneous effects might depend on variations in the oxytocin-receptor gene (*OXTR*). We examined 25 male ASD patients without intellectual disability in a double-blind, cross-over, placebo-controlled fMRI-protocol, in which a single dose of oxytocin or placebo was applied intranasally. Patients performed three experiments in the MRI examining empathy for other’s physical pain, basic emotions, and social pain. All participants were genotyped for the rs53576 single-nucleotide polymorphism of the *OXTR*. Oxytocin increased bilateral amygdala responsiveness during the physical pain task for both painful and neutral stimuli. Other than that, there were no effects of oxytocin treatment. *OXTR* genotype did not significantly interact with oxytocin treatment. Our results contribute to the growing body of empirical literature suggesting heterogenous effects of oxytocin administration in ASD. To draw clinically relevant conclusions regarding the usefulness of oxytocin treatment, however, empirical studies need to consider methods of delivery, dose, and moderating individual factors more carefully in larger samples.

## Introduction

Autism spectrum disorders (ASD) are a group of neuro-developmental disorders characterized by impairments in social communication, social interaction and restrictive, repetitive patterns of behavior^1^. ASD are predominantly genetic in origin, with heritability estimates of around 80%^2^. One symptom reported by individuals with ASD are difficulties in intuitively empathizing with others’ emotional states, especially in situations that require an integration of complex social information^3,4^. These difficulties have been associated with anatomical and functional differences in the empathy network^5–10^. This network, including the anterior cingulate cortex (ACC) and the anterior insula, encodes for both one’s own, as well as others’ bodily, mental, and emotional states^11^. This has led to the idea that humans are able to understand others through an embodied simulation of their own internal states^12^.

Long known for its role in childbirth and lactation^13^, the hormone and neuropeptide oxytocin has also gained attention in the field of psychology and neuroscience as a modulator of various social behaviors^14^. Importantly, intranasal administration of oxytocin has also been found to enhance the ability to share others’ affective and mental states in healthy individuals^15,16^. In this context, the insula^17^ as well as the amygdala seem to be key target regions in the brain, with oxytocin modulating amygdala responsiveness to emotional faces^18–22^ or social stimuli in general^23^. This fits well with findings showing prominent expressions of oxytocin receptors in striatal and limbic regions, such as the amygdala, as well as the ACC^24^, which have strong connections with the frontal cortex and the anterior insula. Further, variations of the oxytocin receptor gene (*OXTR*) have been associated with individual differences in the capacity and tendency to empathize with others’ emotional states in healthy individuals^25–30^ and related brain function in individuals with ASD^31^.

With oxytocin pathways being involved in processes related to sharing others’ emotions, it has been suggested that alterations in the oxytocin system may also be linked to social impairments in ASD. For example, some studies have found lower levels of plasma oxytocin in ASD patients compared to typically developing individuals^32–34^, but results are overall inconsistent^35,36^. Other studies suggest that lower plasma oxytocin levels relate to general social functioning rather than the diagnosis of ASD^35^. Genetic association studies have further linked variation in the *OXTR* with ASD and symptoms in the social domain^37^. Meta-analyses indicated several candidate single nucleotide polymorphisms (SNPs) in the *OXTR* to have significant association both with ASD^38^ and symptom severity^39^. Especially the rs53576 SNP (G > A) has been shown to modulate social cognition both within ASD and the general population^40^. Initial findings suggest that the rs53576 A allele may be a central part of haplotypes associated with ASD^41^, which is in line with studies linking this allele to lower levels of empathy^25^, as well as structural and functional changes in hypothalamus and amygdala in healthy samples^42^. Specifically, it has been shown that A allele carriers have reduced amygdala activation during the processing of faces compared to non-carriers^42^.

Several clinical studies have also investigated the potential of intranasal oxytocin as a treatment for socio-cognitive, behavioral, and socio-affective symptoms of ASD. So far, these studies cannot provide a certain conclusion about oxytocin’s therapeutic potential^43–47^. Results from early studies however suggest that oxytocin increases the attention to social signals and thus may enhance empathy and emotion recognition in individuals with ASD. For example, oxytocin has been shown to increase gazing time on the eye region^33^, and to improve performance on the Reading the Mind in the Eyes Task in adults and adolescents with ASD^48,49^. Imaging studies have provided preliminary evidence that oxytocin influences the ability to understand others’ mental and emotional states by enhancing activation of brain regions within the empathy network, such as the anterior insula^50^. Similarly, oxytocin administration has been shown to increase amygdala activation during face processing specifically in adults with ASD, accompanied by improvements in emotion recognition^51,52^.

However, it has been discussed that the effects of intranasal oxytocin may not be the same for all individuals, but instead depend on stable individual differences, which might reflect variation in the endogenous oxytocin system, including genetic factors^53^. The *OXTR* might be specifically relevant because of its associations with trait empathy and autistic symptoms. Several studies indeed suggest that oxytocin effects on behavior^54–56^ and brain function^57–59^ are modulated by *OXTR* genotype. Considering relevant *OXTR* SNPs could thus be a means of identifying those individuals with ASD that are most responsive to intranasal oxytocin treatment^44,60^, and examining moderating effects of genotype on neural activation as an intermediate phenotype could help to achieve a deeper understanding of how *OXTR* genotypes influence social behavior^14^.

To date, it is still unclear whether *OXTR* genotype moderates the effects of intranasal oxytocin on neural activity when empathizing with others. In the present clinical trial, we used functional magnetic resonance imaging (fMRI) to investigate effects of a single dose of oxytocin on neural correlates of sharing others’ inner states in a sample of young males with ASD without intellectual disability. We focused our analyses on the ACC and anterior insula as parts of the empathy network, as well as the amygdala. Furthermore, to account for individual factors possibly modulating oxytocin effects, we specifically tested the effect of genetic variation on the rs53576 SNP of the *OXTR*. Based on previous findings, we expected increased activation of regions within the empathy network after oxytocin compared to placebo. Further, it was hypothesized that carriers of the risk allele (AA and GA) would show altered activation in empathy-associated networks compared to non-carriers after oxytocin administration.

## Methods

### Patients

Twenty-seven male patients with ASD, aged between 15 and 33 years (*M* = 22.91, *SD* = 4.72 years), were recruited from the Outpatient Clinic for autism spectrum disorders at Marburg University Hospital in Marburg, Germany. All matched the DSM 5 criteria of ASD, had a confirmed ICD-10 diagnosis of autism (*n* = 4) or Asperger’s syndrome (*n* = 23) and had undergone standardized diagnostic procedure at a mean age of 13.1 years (*SD* = 4.73 years) including the Autism Diagnostic Observation Schedule (ADOS^61^), the Autism Diagnostic Interview-Revised (ADI-R^62^) and IQ testing with the Wechsler Intelligence Scale for Children^63^. Current symptom level was assessed with Module 4 of the ADOS at the time of participation (Table 1). Comorbid psychiatric symptoms were assessed using the Diagnostic Interview for Mental Disorders^64^. Patients were not included if they had a verbal IQ below 70, were left-handed or suffered from severe comorbid neurological (e.g. epilepsy), metabolic, endocrinological or cardiovascular conditions. The study was approved by the local ethics committee of the medical faculty of the Philipps-University in Marburg, Germany (214/12 A-ff) and carried out in compliance with the Declaration of Helsinki of 1975, as revised in 2008. All patients received detailed medical information about the study and gave their full informed consent in writing. For patients under the age of 18 years, informed consent was also obtained from their parents or legal guardians. Two patients were excluded after the first study visit because of unexpected clinical findings in one case and subjective negative experiences (nightmares) in another case, leaving data from *n* = 25 patients to be analyzed. Due to technical difficulties during the MRI scan, two patients were excluded from the analyses of the basic emotions experiment, and four were excluded from the analyses of the social pain experiment (see Supplementary Fig. S1 for CONSORT diagram of patient recruitment and study flow).

**Table 1:** Sample characteristics.

	Total sample	Risk allele (AA, GA)	No risk allele (GG)	*p*
	(*N* = 25)	(*n* = 15)	(*n* = 10)	
**At time of study participation**				
Age	22.9 (4.7)	23.1 (3.5)	22.6 (6.3)	0.06
IQ	107.3 (18.1)	111.4 (16.5)	103.4 (20.2)	0.32
AQ	28.6 (9.9)	29.9 (9.5)	26.7 (10.6)	0.44
EQ	25.8 (14.4)	23.6 (12.6)	29.2 (16.8)	0.35
ADOS SA	9.1 (4.4)	9.5 (4.8)	8.6 (4.0)	0.64
ADOS RRB	0.9 (1.0)	0.8 (1.0)	1.1 (1.1)	0.49
ADOS SA + RRB	10.1 (4.6)	10.3 (5.0)	9.7 (4.1)	0.77
ADOS Comparison Score	5.5 (2.6)	5.6 (2.7)	5.3 (2.4)	0.78
Comorbidities (ICD-10 Code)		3 × F3 1 × F42	3 × F3 1 × F42 1 × F40 1 × F90	
**At time of diagnosis**				
Age at time of diagnosis	13.1 (4.7)	13.4 (4.0)	13.1 (5.3)	0.88
ADOS SA	11.5 (3.9)	12.9 (3.7)	9.5 (3.3)	0.03
ADOS RRB	1.9 (1.5)	2.1 (1.6)	1.7 (1.5)	0.50
ADOS SA + RRB	13.5 (4.9)	15.1 (5.1)	11.2 (3.7)	0.06
ADOS Comparison Score	7.3 (2.1)	8.1 (1.9)	6.3 (2.0)	0.03
ADI-R social interaction	17.8 (5.2)	17.6 (5.2)	18.3 (5.5)	0.74
ADI-R communication	13.7 (4.6)	13.4 (3.7)	14.3 (5.6)	0.62
ADI-R stereotyped behavior	5.7 (2.7)	5.6 (3.0)	5.8 (2.1)	0.91

Means, standard deviations (in parentheses) and between-group comparisons on clinical measures in risk allele carriers and non-carriers. *AQ* Autism Spectrum Quotient^65^, *EQ* Empathy Quotient^66^, *ADOS* Autism Diagnostic Observational Schedule^61,67^, *ADOS SA* ADOS Social Affect Score, *ADOS RRB* ADOS Repetitive and Restricted Behavior Score, *ADI-R* Autism Diagnostic Interview-Revised^62^. *p*-values derive from two-sample *t*-tests comparing carriers of the risk allele A (AA, GA) to non-carriers (GG).

### Genotyping for OXTR rs53576

The participants included in this study were drawn from a larger sample of 100 patients with ASD that was analyzed for 22 SNPs in the *OXTR* and its 5′ region including rs53576^68^.

### Clinical trial design

This randomized controlled trial was preregistered at the European Union Clinical Trials Register (EudraCT 2012-003750-89; https://www.clinicaltrialsregister.eu/ctr-search/trial/2012-003750-89/DE; registered 21/11/2012). The trial was stopped prematurely because of poor recruitment (see Supplementary Methods).

This was a monocentric, randomized, double-blind, placebo-controlled, cross-over study. Patients were invited to three visits: an initial visit, including study information, a physical examination, and confirmatory diagnostic procedures regarding the ASD diagnosis, and two MRI sessions that were between 7 and 21 days apart (*Mdn* = 7). Following a medical examination at the beginning of each MRI session, patients received a single dose (24 IU for patients aged ≥ 18 years, *n* = 20; 18 IU for patients between 15 and 17 years, *n* = 5) of oxytocin or placebo intranasally. Patients were randomly assigned to receive oxytocin (*n* = 14) or placebo (*n* = 11) first. 45 min after substance administration they entered the MRI and performed three consecutive experiments. No adverse side-effects of the nasal spray were observed. For details on the assessment of side effects see Supplementary Methods and Supplementary Tables S7 and S8.

### Stimuli and paradigms

**Figure 1:**
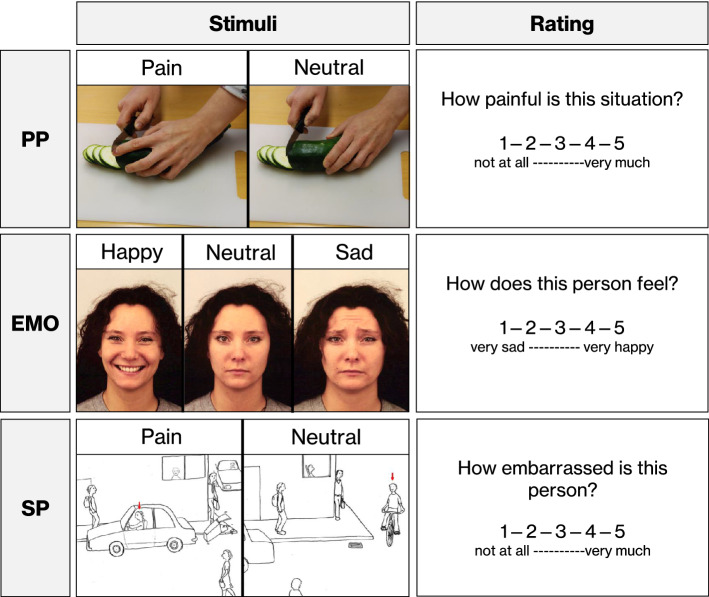
Stimuli and rating questions of the three experiments. In all three experiments, patients were required to view stimulus pictures and respond to a subsequent rating question. The upper row shows two example stimuli of the physical pain (PP) experiment, reflecting the two stimulus categories used in the experiment, and the respective rating question. Example stimuli and rating questions for the basic emotions (EMO) and social pain (SP) experiments are displayed in the middle and lower row. Stimuli for the basic emotions experiment were taken from the Karolinska Directed Emotional Faces database^69^, which is publicly available and may be used for non-commercial scientific research purposes (https://www.kdef.se/; stimuli shown here: BF02HAS, BF02NES, BF02SAS). In the physical pain and basic emotions experiment, patients were instructed to look at the photograph for 4.5 s and to rate the intensity of pain or the quality of emotion that the depicted person would experience. In the social pain experiment, sketches showed a protagonist, indicated by a red arrow above their head, in potentially embarrassing (socially painful) or neutral situations. Each sketch was accompanied by a sentence introducing the current scenario [for these specific situations: “You are on the pavement: a car driver knocks over a trash can while backing into a parking space.” (Pain); “You are in the city: a cyclist turns right.” (Neutral)]. Patients were instructed to look at each sketch for 12 s and to subsequently rate the intensity of the embarrassment the protagonist would feel in the respective scenario. The length of the rating phase was 3 s in all experiments.

The fMRI experiments examined participants’ evaluations of and neural responses to others’ social pain, expression of basic emotions and physical pain (see Fig. 1 for stimulus examples, rating questions and timings). The experiments were conducted in exactly this order, using parallel versions during the first and second MRI session. In all experiments, participants viewed pictures that were presented to them on a screen. In the social pain task, pictures were hand-drawn sketches showing a person in an embarrassing (social pain, SP) or neutral (social neutral, SN) social situation. In the basic emotions task, patients viewed photographs of faces displaying a happy (HAP), sad (SAD) or neutral (EN) expression. In the physical pain task, pictures showed a person’s hand or foot in a painful (physical pain, PP) or non-painful (physical neutral, PN) situation. After each picture, participants were required to rate the intensity of pain or quality of emotion the depicted person would experience in the scenario (1 = no pain/very sad, 5 = very strong pain/very happy) within a 3-s time window using a button press of the right hand (see Supplementary Methods for task details). In total, the social pain task lasted 16 min, the basic emotions task lasted 8 min and the physical pain task lasted 8 min.

### Functional MRI data acquisition

Participants were scanned at 3 Tesla (Siemens Magnetom Trio, Erlangen, Germany) with 36 near-axial slices and a distance factor of 10% providing whole brain coverage. For the acquisition of functional images, an echo planar imaging (EPI) sequence was used (TR = 2.2 s, TE = 30 ms, flip angle = 90, slice thickness = 3 mm, FoV = 190 × 190 mm). In total, 402 functional images were obtained for the social pain experiment, 218 images were obtained for the basic emotions experiment, and 210 images were obtained for the physical pain experiment. To rule out potential anatomical abnormalities, high resolution images with a T1-weighted 3D MP-RAGE sequence were acquired (TR = 1900 ms, TE = 2.52 ms, number of slices = 176 (sagittal), slice thickness = 1 mm, FoV = 256 × 256 mm, voxel size = 1 × 1 × 1 mm).

### Data analysis

#### Behavioral data

Behavioral data analysis was conducted using IBM SPSS Statistics 22 (Armonk, NY: IBM Corp. Released 2013). To examine the effects of treatment on stimulus ratings, we calculated two-way repeated-measures ANOVAs for each experiment, including treatment (oxytocin, placebo) and stimulus category as within-subject factors. Further, genotype was added as a covariate. We applied an allele-load model and coded genotype in a linear fashion (AA < GA < GG), since trait empathy has been found to vary as a function of the number of risk alleles on the rs53576 SNP^29^. For additional analyses examining genotype-treatment interactions on differential behavioral responses during the three experiments, see Supplementary Information. Paired t-tests were calculated to assess mean rating differences of oxytocin and placebo within each stimulus category. P-values were corrected for multiple comparisons using the Holm–Bonferroni method^70^.

To explore possible interactions of treatment with participant age, received dose (18 vs. 24 IU, depending on age), and order of treatment (oxytocin first, placebo first), we calculated three additional repeated-measures ANOVAs per experiment, each with stimulus category and treatment as within-subject factors, genotype as covariate, and age, dose or order as additional covariate or between-subjects variable.

#### Functional MRI data

Functional MRI data were preprocessed and analyzed using the statistical parametric mapping software package (SPM12, retrieved from www.fil.ion.ucl.ac.uk/spm). For each session, the volumes were corrected for slice timing, head motion, and spatially normalized to the Montreal Neurological Institute standard space by applying non-linear transformations obtained from a segmentation of the mean EPI image. The normalized volumes were resliced with a voxel size of 2 × 2 × 2 mm, smoothed with an 8 mm full-width half-maximum isotropic Gaussian kernel and high-pass filtered with a cutoff period of 128 s. Statistical analyses were then performed using a mixed-effects, two-level procedure for each experiment.

For every fMRI experiment, we used analog procedures in the first- and second-level analyses. In the first-level analysis conducted for each subject and session, we included regressors modeling the hemodynamic responses to each stimulus category (i.e., SP and SN for social pain, HAP, SAD and EN for basic emotions and PP and PN for physical pain), as well as the rating period. Six parameters modeling head motion on a scan-to-scan basis were added as regressors to account for noise. The resulting β-maps of activation during each stimulus category were used in the analysis on the second level. Here, we defined random-effects general linear models with the two within-subject factors treatment (oxytocin, placebo) and stimulus category. Genotype was included as a covariate interacting with treatment. For additional analyses examining genotype-treatment interactions on responses within the regions of interest, see Supplementary Information. To explore potential moderating effects of age, dose, and order, we defined three additional general linear models per experiment including the covariate of interest in interaction with treatment. All fMRI results were family-wise error (FWE)-corrected for multiple comparisons on the voxel-level in both whole-brain and regions of interest (ROI) analyses.

### Regions of interest analyses

**Figure 2:**
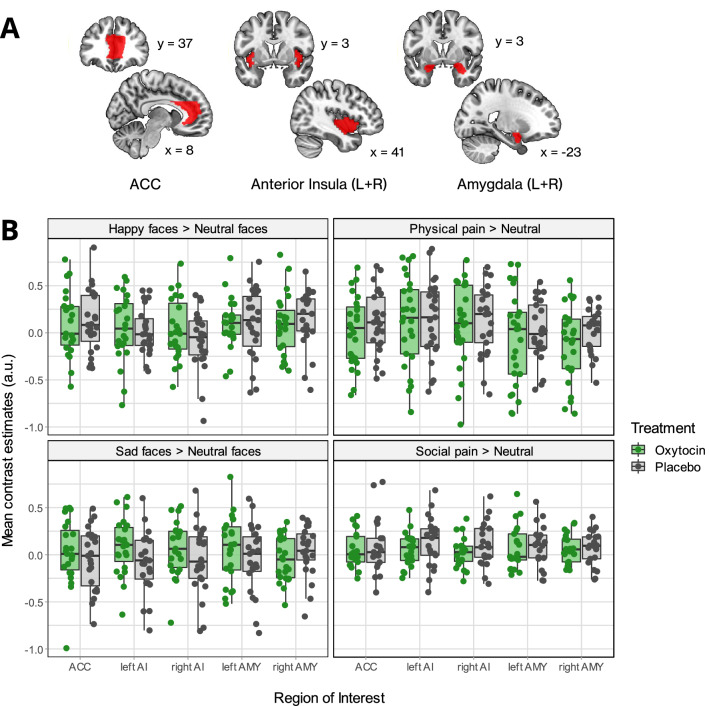
Regions of Interest and task-related mean activations during all experiments. A. Size and location of predefined Regions of Interest (ROIs). ACC = Anterior cingulate cortex. Images created with MRIcroGL (https://www.nitrc.org/projects/mricrogl/). B. Mean contrast estimates for all ROIs, displayed separately for each examined contrast. *AI* anterior insula, *AMY* amygdala, *a.u.* arbitrary units. Data derived from *N* = 25 patients for the physical pain experiment, *N* = 23 for the basic emotions experiment, and *N* = 21 for the social pain experiment. Plots were created using the package ggplot2 in R^77,78^.

Because of the pivotal role of the ACC and anterior insula in the experience of physical and social pain^71–73^ and the amygdala being a target region for oxytocin effects^16,18,52^, we used anatomically defined ROIs in all three experiments to constrain the search space to these key areas. Masks of the ACC and amygdala were created using the automated anatomic labeling atlas (AAL)^74^ integrated in the WFU PickAtlas^75^. Since the AAL does not differentiate anterior and posterior sections of the insula, we used a validated three-cluster parcellation scheme to obtain a valid anterior insula mask^76^. We chose two clusters specified as dorsal and ventral anterior insula and merged them to a single mask for left and right anterior insula, respectively. All ROI analyses were conducted using the small-volume correction as implemented in SPM12. Location and shape of all ROIs are displayed in Fig. 2A.

## Results

### Physical pain

#### Behavioral data

Mean stimulus ratings after oxytocin and placebo are shown in Table 2. Participants with ASD clearly distinguished between PP and PN, as indicated by a significant main effect of category on stimulus ratings (*F*(1,23) = 901.89, *p* < 0.001, partial η^2^ = 0.975). There were no significant main effects of treatment or *OXTR* genotype, and no significant interaction effects.

**Table 2:** Summary of behavioral data.

			Oxytocin		Placebo		*p'*	*d*
			*M*	*SD*	*M*	*SD*		
Ratings	Physical pain	PP	3.91	0.47	3.94	0.45	1.000	0.07
		PN	1.25	0.33	1.11	0.16	0.406	− 0.54
	Basic emotions	SAD	1.62	0.25	1.49	0.31	0.429	− 0.46
		HAP	4.44	0.40	4.54	0.40	1.000	0.25
		EN	2.77	0.21	2.82	0.22	1.000	0.23
	Social pain	SP	4.04	0.42	3.99	0.34	1.000	− 0.13
		SN	1.22	0.37	1.12	0.21	1.000	− 0.33
RTs (ms)	Physical pain	PP	765	254	737	267	1.000	− 0.11
		PN	698	248	694	239	1.000	− 0.02
	Basic emotions	SAD	873	305	774	264	0.492	− 0.35
		HAP	737	244	697	272	1.000	− 0.16
		EN	706	269	709	253	1.000	0.01
	Social pain	SP	974	328	972	410	1.000	− 0.01
		SN	892	280	774	305	0.726	− 0.40

*p*-values derive from paired *t*-tests comparing mean ratings after oxytocin and placebo administration. Values in the *p*′-column represent *p*-values adjusted for multiple comparisons using the Holm-Bonferroni correction while keeping the significance level of *p* < 0.05 constant. *p′*-values of 1.000 denote upper bound estimates. Data derived from *n* = 25 patients for the physical pain experiment, *n* = 23 for the basic emotions experiment, and *n* = 21 for the social pain experiment.

*RT* Reaction time, *PP* physical pain, *PN* physical neutral, *SAD* Sad, *HAP* Happy, *EN* emotional neutral, *SP* social pain, *SN* social neutral, *M* mean, *SD* standard deviation.

Controlling for age, dose, and order of treatment did not change the magnitude of these effects (Supplementary Table S10). However, age was positively associated with mean stimulus ratings (*F*(1,22) = 6.49, *p* = 0.018, partial η^2^ = 0.228). A post-hoc correlation analysis indicated that older participants gave higher mean ratings of physical pain regardless of stimulus category (*r*(23) = 0.49, *p* = 0.012). Further, there was a significant treatment × order interaction (*F*(1,22) = 10.95, *p* = 0.003, partial η^2^ = 0.332). Post-hoc t-tests suggested that this interaction was driven by the group that received oxytocin first, where average ratings where lower after oxytocin compared to placebo (mean difference = 0.17, *t*(22) = 3.26, *p*(corrected) = 0.021, *d* = 0.83).

#### Functional MRI data

Whole brain analyses indicated that PP, compared to PN, induced strong and consistent activations of brain regions that have been previously implicated in empathy for or others’ physical pain^79^, including ACC, anterior insula and supramarginal gyrus (Supplementary Table S5, Fig. 2B). Treatment effects were examined using the contrasts oxytocin > placebo, placebo > oxytocin and in interactions with stimulus category. On the whole-brain level, oxytocin was found to enhance average activation within the right amygdala and inferior temporal gyrus (k = 11, MNI coordinates: 34/0/− 26, *t*(94) = 5.12, *p*_*FWE*_ = 0.017), and left hippocampus (k = 10, − 34/− 8/− 22, *t*(94) = 5.10, *p*_*FWE*_ = 0.019) compared to placebo. ROI analyses revealed that oxytocin also increased average left amygdala activation (k = 29, − 22/− 2/− 12, *t*(94) = 3.95 *p*_*FWE*_ = 0.007), although this effect was more pronounced in the right amygdala (k = 131, *p*_*FWE*_ < 0.001). There were no significant interactions of stimulus category and treatment, and no main effects of *OXTR* genotype, both in whole-brain and ROI analyses. Whole-brain analyses however showed significant genotype-treatment interactions in the left precuneus (k = 50, − 2/− 46/10, *t*(94) = 5.68, *p*_*FWE*_ = 0.002) and white matter within the left frontal inferior orbital region (k = 10, − 24/30/− 6, *t*(94) = 5.42, *p*_*FWE*_ = 0.006). Here, genotype was more strongly associated with activation under placebo conditions than after oxytocin.

Controlling for age, dose, and order of treatment did not change the magnitude of these effects (Supplementary Fig. S2). Regardless of stimulus category or treatment, age was significantly correlated with activation of a small cluster within the left superior parietal lobe (k = 5, − 30/− 54/58, *F*(1,92) = 30.04, *p*_*FWE*_ = 0.009). Age, dose, and order did not significantly interact with treatment.

### Basic emotions

#### Behavioral data

A main effect of category was observed, indicating that patients clearly distinguished the emotional expressions of the shown faces (*F*(2,42) = 486.8, *p* < 0.001, partial η^2^ = 0.959). Further, the interaction of category and treatment was significant (*F*(2,42) = 8.10, *p* = 0.001, partial η^2^ = 0.278). However, post-hoc comparisons could not detect significant mean differences between ratings after oxytocin and placebo when correcting for multiple comparisons (Table 2). There were no significant main effects of treatment or *OXTR* genotype, and no significant interaction effects.

When controlling for age, the treatment × category effect was no longer significant, and there was a significant main effect of genotype on mean ratings when controlling for dose (*F*(1,20) = 4.75; *p* = 0.041, partial η^2^ = 0.192). Otherwise, controlling for age, dose and order did not influence treatment effects on ratings (Supplementary Table S11). There was a main effect of dose on mean stimulus ratings (*F*(1,20) = 7.65, *p* = 0.012, partial η^2^ = 0.277; 18 IU: *M* = 3.03, *SD* = 0.094, *N* = 5; 24 IU: *M* = 2.93, *SD* = 0.110, *N* = 18). Age, dose, and order did not significantly interact with treatment.

#### Functional MRI data

The facial stimuli induced widespread activity within occipital regions, including the fusiform and occipital face area, as well as inferior frontal regions, anterior insula, and medial cingulate cortex (conjunction analysis of SAD, HAP and EN, Supplementary Table S6, see Fig. 2B for difference contrasts between emotional and neutral stimuli). Treatment effects were examined using the contrasts of oxytocin > placebo, placebo > oxytocin and in interactions with affective facial expression. There were no effects of treatment in both whole-brain analyses and ROI analyses. Whole-brain analyses showed that on average, genotype was significantly associated with a small cluster within the right primary visual cortex (k = 2, 20/− 100/0, *F*(1,130) = 25.21, *p*_*FWE*_ = 0.031).

When controlling for age, the effect of genotype was no longer significant. When controlling for dose, the genotype effect was significant in two additional small clusters (ks ≤ 5) in the left fusiform gyrus (− 28/− 76/− 16, *F*(1,128) = 30.18, *p*_*FWE*_ < 0.001) and right lingual gyrus (16/− 84/− 6, *F*(1,128) = 26.97). Otherwise, controlling for age, dose, and order of treatment did not change the magnitude of the effects (Supplementary Fig. S3). On average, age was associated with activation within the right middle occipital gyrus (k = 25, − 18/− 100/2, *F*(1,128) = 36.89, *p*_*FWE*_ < 0.001), and dose was associated with activation within left middle occipital gyrus (k = 66, − 42/− 80/− 2, *F*(1,128) = 42.12, *p*_*FWE*_ < 0.001) and right lingual gyrus (k = 29, 16/− 82/− 6, *F*(1,128) = 35.25, *p*_*FWE*_ = 0.001). Age, dose, and order did not significantly interact with treatment.

### Social pain

#### Behavioral data

Empathy for others’ social pain was successfully induced, as shown by a significant main effect of category (*F*(1,19) = 1413.25, *p* < 0.001, partial η^2^ = 0.987). There were no significant main effects of treatment or *OXTR* genotype, and no significant interaction effects. Controlling for age, dose, and order of treatment did not change the magnitude of these effects (Supplementary Table S12).

#### Functional MRI data

Whole-brain analyses revealed that SP, compared to SN, elicited significant activation in posterior temporal regions, posterior medial frontal gyrus, thalamus, inferior frontal gyrus, and supramarginal gyrus (Supplementary Table S7, Fig. 2B). Treatment effects were examined using the contrasts of oxytocin > placebo, placebo > oxytocin and in interactions with stimulus category. Again, there were no significant treatment effects in both whole-brain analyses and ROI analyses, and no significant effects of genotype or genotype-treatment interactions.

When controlling for dose, genotype was on average significantly associated with activation within the right lingual gyrus (k = 43, 16/− 84/− 6, *F*(1,116) = 34.49, *p*_*FWE*_ = 0.001). Otherwise, controlling for age, dose or order did not change the magnitude of effects (Supplementary Fig. S4). On average, age was significantly associated with activation in a cluster within the left inferior occipital gyrus (k = 8, − 36/− 90/− 10, *F*(1,116) = 32.73, *p*_*FWE*_ = 0.002). Age, dose, and order did not significantly interact with treatment.

## Discussion

The present study examined the influence of acute oxytocin administration on neural correlates of empathy in individuals with ASD and how these effects are modulated by *OXTR* genotype. While all three tasks elicited activation within networks related to sharing another person’s affective state, our findings are inconclusive regarding the effects of oxytocin on brain activation. Of all analyses across three experiments, including analyses restricted to anatomically defined search areas, we found only one significant effect of oxytocin on brain activation. All other comparisons yielded non-significant results. *OXTR* genotype did not significantly influence oxytocin efficacy in the hypothesized regions. Exploratory analyses showed that controlling for participant age, received dose and order of treatment administration did not influence treatment effects.

Overall, our results are part of a growing number of studies that report heterogeneous effects of intranasal oxytocin in the context of ASD. While most randomized clinical trials have found some oxytocin effects on ASD symptoms^33,50–52^ and brain function^50–52,80,81^, not all trials have reported effects^82^. Importantly, our findings are in line with recent meta-analytical evidence indicating that intranasal oxytocin only leads to small but non-significant changes in overall social cognition in ASD^44^, but has no significant effect on emotion recognition and empathy^83^.

The only significant oxytocin effect on brain activation was found during the physical pain experiment, where oxytocin, compared to placebo, enhanced activation in bilateral amygdala and left hippocampus, irrespective of whether the pictures showed painful scenes or not. While this finding fits with previous reports that oxytocin administration leads to an increase in amygdala activation during the processing of faces in men with ASD^51,52^, these findings are not consistent with studies that examine neural responses to aversive stimuli in men without an ASD diagnosis. These studies have repeatedly demonstrated decreased amygdala activation, which has been interpreted as a neural mechanism underlying oxytocin’s anxiolytic effects^18,20,21^. Further, our result of increased amygdala activation is not in line with previous studies examining empathy for others’ physical pain in men without ASD, showing reduced activation in the left insula after oxytocin administration^84^. The increase in amygdala reactivity during the physical pain task might be specific to ASD and reflect increased sensitivity in a context involving the potential injury to another person, regardless of whether the injury actually occurs. This would however suggest that oxytocin leads to a less differentiated response to the pain or absence of pain in others, which might in fact reflect a less empathic state than under placebo conditions. However, given that the effects on the brain level were not associated with robust behavioral effects, and that this was the only significant oxytocin effect found among the multitude of contrasts examined within this study, this interpretation should be considered speculative.

In principle, there are several possible explanations for the predominant absence of general oxytocin effects throughout the remaining analyses. Besides concerns relating to the intranasal method of administration and optimal doses of oxytocin^85^, reasons might lie in the specificity of our sample. First, given our sample size of 25 analyzed datasets, the current study did not have sufficient statistical power to reliably detect possible small effects of intranasal oxytocin. It is therefore unclear whether our findings reflect true null effects or if they are the consequence of data insensitivity. However, it must be noted that our sample is larger than the average sample size of previous studies examining oxytocin effects on brain function in ASD^44^, and comparable to the average sample size in clinical trials examining oxytocin effects across different neurodevelopmental disorders^83^. Moreover, previous studies showing oxytocin effects on emotion recognition, empathy, or face processing in adults with ASD had an average sample size of 21.5 participants^48,50,51,86–88^. If the effects found in these studies are true effects with roughly accurate effect size estimates, we would expect to find them even within in our sample. Second, the sample was relatively homogeneous, including only males without intellectual disability and with moderate levels of symptomatology at the time of data collection, which cannot be considered representative for the heterogeneous phenotype of ASD.

Moreover, all participants had received multiple interventions since their initial diagnosis in childhood. Interestingly, carriers of the rs53576 A allele showed higher levels of ASD symptomatology in their childhood compared to non-carriers, but both groups displayed comparable symptom levels at the time of study participation. Possibly, our participants, and especially A allele carriers, might have benefitted from these interventions at such a rate that a ceiling effect was reached for oxytocin efficacy. Further, epigenetic processes might have influenced the sensitivity of A-allele carriers to intranasal oxytocin. There is evidence that the A allele of rs53576 is linked to increased methylation levels of CpG-islands of the *OXTR*^89,90^ and reduces the *OXTR* gene expression in distinct brain regions in individuals with ASD^91^. In the same way as allele-specific DNA-methylation might mask or reveal associations between *OXTR* rs53576 genotype and phenotype^92^, it might also impact the effect of intranasal oxytocin on social cognition.

Indeed, we did not find influences of *OXTR* rs53576 genotype on oxytocin effects in this study. This is certainly related to the limited statistical power of our sample, also given the low frequency of homozygous carriers of the A allele. Further, it is more likely that a combination of *OXTR* SNPs and OXTR haplotypes, rather than a single SNP, modulates social-cognitive abilities^93^. Considering multiple *OXTR* SNPs could thus be a way of identifying individuals who benefit most of intranasal oxytocin in future research, if at any point in the future sample sizes provide sufficient power to detect these effects.

It has been frequently discussed that oxytocin effects are influenced by trait-level individual differences and underlying variation in the endogenous oxytocin system^53^. Although this study explicitly examined potential moderating influences of *OXTR* genotype, other potential moderating factors could not be addressed, which might have led to overall non-significant effects. For example, the age range in this sample was 15–33 years, with a significantly larger proportion of adults (18 years or older). Since oxytocin has been shown to influence brain and behavior differently at different developmental stages^94^, oxytocin efficacy might differ between adults and adolescents. Although controlling for the received dose and age did not change the direction and magnitude of our main results, future studies could examine the influence of age in larger and more balanced groups to obtain higher statistical power. Further, after the preregistration of this study, several studies have been published showing that social anxiety and baseline plasma oxytocin levels can influence oxytocin efficacy in autistic individuals^87,95,96^. Since we did not measure baseline oxytocin levels or anxiety, we cannot rule out moderating effects of these factors in our sample. However, these effects are still not well understood, and more recent evidence has raised doubts on whether plasma oxytocin levels are significantly related to brain levels under baseline conditions^97^.

An alternative explanation for the absence of a general treatment effect in this study relates to an overestimation of effect sizes of intranasal oxytocin treatment based on earlier studies. It has been criticized that most studies examining oxytocin effects have too small sample sizes to even detect medium effects and are therefore substantially underpowered^98^. Generally, effect sizes of studies using intranasal oxytocin seem to be smaller than initially assumed^99^, a finding that is also reflected in unsuccessful attempts to replicate earlier findings of oxytocin effects on trust^100,101^ and mind-reading in healthy populations^102^. Moreover, biases in reporting and publishing results may have contributed to an overestimation of effect sizes in human oxytocin research. While there is some evidence that publication bias is present in the broader field of human oxytocin studies^103^, meta-analyses examining publication and researcher bias in oxytocin studies in the context of ASD are rather inconclusive^44,83^.

The variety of findings of human oxytocin studies have led to several notions about how oxytocin generally influences social behavior, cognition, and emotion. While some hypothesize that oxytocin specifically targets social processes^104^, other models suggest a more general function, such as anxiety reduction^105^, regulation of approach/avoidance motivation^106^ or allostasis^107^. In this context, it is difficult to predict how and if intranasal oxytocin affects social cognition and social affect in ASD. However, to determine oxytocin’s therapeutic potential, there is a need for a basic understanding and robust and replicable measures of its effectiveness. In the long run, this can only be achieved by focusing on core psychological processes related to ASD symptoms and using standardized protocols in multiple, heterogeneous samples that can in the end be collapsed to reach enough power. Encouraging replication studies will enable researchers to achieve a nuanced understanding of how and if oxytocin effectiveness is moderated by biological factors (e.g. genetic variation, brain development and endogenous oxytocin levels), personality traits, symptom severity and comorbidities, and which specific psychological processes are targeted under which circumstances. Whether or not oxytocin nasal spray may benefit individuals with ASD, either as a discrete treatment option or in combination with behavioral therapy, cannot be assessed to date.

## Conclusions

In conclusion, our study does not provide support for effects of a single dose of oxytocin on neural processes underlying an affective route to understanding others in males with ASD without intellectual disability. While variation of the rs53576 SNP on the *OXTR* did not influence the efficacy of oxytocin in our sample, genetic modulation of oxytocin effects on empathy cannot be ruled out. The effects of a single dose of intranasal oxytocin might have been too small to lead to a detectable change in brain function and affective experience in our sample of 25 individuals, which might suggest that its use as a therapeutic intervention alone may be limited. An in-depth understanding of how oxytocin is relevant to the heterogenous phenotype of ASD can be achieved by considering moderating individual factors, using paradigms capturing clinically relevant psychological processes in sufficiently large samples, and reporting findings in an unbiased fashion.

## Supplementary Information

Supplementary Information.

## Data Availability

The datasets generated and analyzed during the current study are not publicly available due to them containing information that could compromise research participant privacy and consent. Fully anonymized data are available from the corresponding author on reasonable request.

## Abbreviations

AAL: Automated anatomic labeling
ACC: Anterior cingulate cortex
ADI-R: Autism diagnostic interview-revised
ADOS: Autism diagnostic observation schedule
ASD: Autism spectrum disorders
EN: Emotional neutral faces (task condition)
EPI: Echo planar imaging
fMRI: Functional magnetic resonance imaging
FWE: Family-wise error (correction)
HAP: Happy faces (task condition)
*OXTR*: Oxytocin receptor gene
PN: Physical neutral (task condition)
PP: Physical pain (task condition)
ROI: Regions of interest
SAD: Sad faces (task condition)
SN: Social neutral (task condition)
SNP: Single nucleotide polymorphism
SP: Social pain (task condition)
