# Supplementary material for: Randomized clinical trial shows no substantial modulation of empathy-related neural activation by intranasal oxytocin in autism
Source: Sci Rep. 2021 Jul 23;11:15056. doi: 10.1038/s41598-021-94407-x (PMC8302641; doi:10.1038/s41598-021-94407-x)
Supplement: Supplementary file 1 — Supplementary Information. [file 41598_2021_94407_MOESM1_ESM.pdf]

SUPPLEMENTARY INFORMATION

For

Randomized clinical trial shows no substantial modulation of empathy-related neural activation by intranasal oxytocin in autism

Annalina V. Mayer*, Anne-Kathrin Wermter*, Sanna Stroth, Peter Alter, Michael Haberhausen, Thomas Stehr, Frieder M. Paulus, Sören Krach, Inge Kamp-Becker

Correspondence to Annalina V. Mayer: ann.mayer@uni-luebeck.de

SUPPLEMENTARY METHODS

Intended and actual sample size

Sample size calculations were carried out using G*Power[1]. We calculated the minimum number of participants that would be necessary to identify a significant interaction between genotype and treatment (placebo/oxytocin) in a repeated-measures ANOVA ($\alpha = 0.001$ and two groups (A/A + A/G and G/G)). We expected the effects of oxytocin to be of medium to large size ($f = 0.32$, corresponding to a *d*-value of 0.64). The study was planned and pre-registered when effect sizes of intranasal oxytocin treatment in ASD were largely unknown. Existing studies however suggested relatively large effects especially in ASD patients, with a meta-analysis reporting a combined effect size of $d = 0.57$[2]. Assuming a correlation between both measurements of $r = .50$, a sample size of $N = 58$ (29 per genotype subgroup) would ensure sufficient statistical power of 90%. With an expected drop-out rate of 20%, which had proved plausible in the past, the overall sample size was calculated to be $N = 72$ (36 participants per subgroup).

The intended sample size was not achieved within the planned study period and the study was ended prematurely. The reason for this were significant time delays in conducting the clinical trial and difficulties with patient recruitment. The study could not be carried out with patients within the initially planned age range of 12-35 years due to a decision by the German Federal Institute for Drugs and Medical Devices (BfArM). Since we intended to recruit participants from an existing pool of genotyped children, adolescents and young adults with ASD, and many of these were children between 12-14 years, this decision restricted our potential sample size. We contacted all age-eligible male patients within the existing database (around 140), of which 41 were screened. As genotyping was a prerequisite for participation in the study, it was not possible to recruit many more participants outside of this database, even though we were able to genotype a few additional patients ($n = 2$). When all possibilities of recruitment within the existing database were exhausted and there was no prospect of further participants in the foreseeable future, we decided to terminate the study prematurely. This decision was made when the planned timeframe for data collection had already been exceeded by 6

months. Eventually, of a total of 41 patients screened, 28 were randomized and data from 25 patients could be analyzed.

Patient recruitment and inclusion/exclusion criteria

Patients were recruited from January 2015 to June 2017 at the Outpatient Clinic for Autism Spectrum Disorders at Marburg University Hospital in Marburg, Germany, where they had received a diagnosis of ASD prior to study participation. Eligible patients and their families were contacted through an invitation letter including brief information on the study. We invited male patients aged 15 to 35 years who had previously partaken in a molecular genetic study at the same study site, during which they had been genotyped for the rs53576 SNP of the *OXTR*. All were German native speakers. Concurrent psychopharmacological treatment was not an exclusion criterion, however, patients receiving medication (antidepressants, $n = 1$) were required to keep the dosage constant for the entire period of data acquisition. Principal exclusion criteria were a body mass index less than 18 or higher than 30, verbal IQ below 70 as measured with the Wechsler Intelligence Scale for Children[3], traumatic lesions of the brain, severe neurological disorders (e.g. epilepsy), metal implants (MRI contraindication), acute suicidal tendency according to the clinical impression, known metabolic or endocrinological disorders, cardiac disorders (assessed through anamnesis, electrocardiogram, blood pressure and heart rate measurements), hypersensitivity to nasal sprays or other drugs, and comorbid drug, alcohol or nicotine (more than 15 cigarettes per day) abuse or dependence.

Randomization and blinding

Randomization (simple randomization) was carried out independently from the recruitment process and data acquisition by the Coordination Center for Clinical Studies at Philipps-University (KKS) Marburg, which created a list of subject numbers (“randomization numbers”) for each genotype subgroup (carriers vs. non-carriers of the risk allele). Each number was thereby randomly assigned to an administration sequence (oxytocin first, placebo first). Blinding was carried out by an independent pharmacist at the Clinic Pharmacy of Heidelberg University Hospital, who received the complete list of randomization numbers and corresponding administration sequence from the KKS Marburg. Oxytocin and placebo were filled into spray bottles of identical appearance. Two bottles (one with oxytocin and one with placebo) were sealed together in a secondary packaging (foil). Secondary packaging and spray bottles were each provided with a specific randomization number. The bottles were additionally labeled with the sequence of administration (1 or 2). The packaged study drugs were sent to the trial center at the Department of Child- and Adolescent Psychiatry of Philips-University Marburg, where the patients were enrolled. After a study physician had obtained the patient’s informed written consent, the KKS was contacted, which then assigned a randomization number to the patient. All investigators engaged in recruitment, data acquisition (including study nurses, study physicians and research assistants), and data analysis, as well as the patients, were blinded to the allocation arm, i.e. the administration sequence.

Procedure during study visits

MRI scanning took place at the brain-imaging unit at the Department of Psychiatry at Phillips-University in Marburg, Germany. Patients were invited to an initial visit that included study information, a physical examination, and confirmatory diagnostic procedures regarding the ASD diagnosis. Patients who met all inclusion criteria were then invited to two MRI sessions. These sessions were planned to be 7 days apart, which was achieved for most patients (n = 17). If this was not possible, the second session took place within 21 days after the first session. Patients were instructed not to eat, smoke or drink excessive amounts of water two hours before administration of the nasal spray. In the beginning of the first MRI session, contraindications for oxytocin (cardiac arrhythmia) were excluded by performing an auscultation of the heart and an ECG. During both MRI sessions, each participant's medical history, current health status, heart rate and blood pressure were assessed. Following the medical examination, a study physician administered the nasal spray to the patients. Six puffs per nostril (24 IU) were administered to patients aged 18 years or over, and four and five puffs (18 IU) were administered to the right and left nostril, respectively, in patients aged 15-17 years. The nasal spray contained either oxytocin (Syntocinon-Spray®; each puff containing 2 IU of oxytocin) or placebo (containing all ingredients except for the peptide). The placebo nasal spray was identical to the oxytocin nasal spray in appearance and smell.

Possible adverse effects were assessed using the Multidimensional Mood Questionnaire[4], the Self-Assessment Manikin[5], and a questionnaire asking about any subjectively experienced side-effects, as well as by monitoring heart rate and blood pressure three times before and once after the MRI measurement. Patients did not report any side effects during this trial. For analyses of treatment effects on mood and affective states please see Supplementary Tables S7 and S8.

fMRI paradigms

In the current study, we used three experiments examining the neural correlates of sharing others' mental and affective states. Two of these experiments, the physical and social pain experiment, have been previously described and applied in samples of autistic males without intellectual disabilities[6–10].

In the physical pain experiment, 28 digital color photographs were presented, depicting another person's left or right hand or foot from a first-person perspective in either painful (physical pain, PP) or nonpainful (physical neutral, PN) situations[11]. Painful and neutral stimuli were matched in number as well as for semantic content and luminance. In each trial, patients were instructed to look at the photograph for 4.5 seconds and to rate the intensity of pain that the depicted person would experience in the respective scenario (1 = no pain, 5 = very strong pain). A fixation cross followed the rating phase of 3 seconds for an average of 6.1 seconds.

In the basic emotions experiment, 30 digital color photographs were presented[12], showing a male or female face with a happy (HAP), sad (SAD) or emotionally neutral (EN) expression (10 photographs per stimulus category). Patients were instructed to look at each stimulus for 4.5 seconds and to rate

how the depicted person felt (1 = very sad, 5 = very happy). A fixation cross followed the rating phase of 3 seconds for an average of 6.1 seconds.

In the social pain experiment, overall 36 validated hand-drawn sketches were presented, each displaying a marked protagonist in an either potentially embarrassing (social pain, SP, 12 sketches) or neutral public scenario (social neutral, SN, 12 sketches; another 12 sketches displaying a protagonist in an inappropriate scenario that he/she is not aware of[13] were not included in the current analysis). The sketches were presented together with a brief description of the situation. Participants were instructed to look at each sketch for 12 seconds and to subsequently rate the intensity of the embarrassment the protagonist would feel in the respective scenario (1 = no embarrassment, 5 = very strong embarrassment). A blank screen with a fixation cross (1 second) was interleaved between the presentation of the sketch and the rating period (3 seconds), which was followed by an 8-second low-level baseline separating the trials.

fMRI analyses

Physical Pain: second-level analysis

For each subject and session, we included three regressors modeling the hemodynamic responses to physical pain (PP) and the corresponding neutral (PN) stimuli, as well as the rating period, with durations as described above. Six parameters modeling head motion on a scan-to-scan basis were added as regressors to account for noise. The resulting individual β-maps of activation during PP and PN were used in the analysis on the group level. Here, we defined a random-effects GLM with two factors (2x2) to investigate effects of treatment (oxytocin, placebo as a within-subject factor) and stimulus category (PP, PN as a within-subject factor) on activation differences. We included the order of treatment (oxytocin first, placebo first) as a between-subject factor to control for possible order effects.

Basic Emotions: second-level analysis

On the individual subject level, the GLM included four regressors for the sad (SAD), happy (HAP) and neutral (EN) facial stimuli, as well as the rating period, with durations described above. Again, six parameters modeling head motion on a scan-to-scan basis served as additional regressors to account for noise. The resulting β-maps of activation during SAD, HAP and EN were used in the analysis on the group level. Here, we defined a random-effects GLM with two factors (2x3) to investigate effects of treatment (oxytocin, placebo as a within-subject factor) and stimulus category (SAD, HAP, EN as a within-subject factor) on activation differences. Again, the order of treatment was included to control for possible order effects.

Social Pain: second-level analysis

On the individual subject level, the GLM included three regressors for social pain (SP) and neutral (SN), as well as the rating period, with durations described above. Again, six parameters modeling head motion on a scan-to-scan basis served as additional regressors to account for noise. The resulting β-maps of activation during SP and SN were used in the analysis on the group level. Again,

we defined a random-effects GLM with two factors (2x2) to investigate effects of treatment (oxytocin, placebo as a within-subject factor) and stimulus category (SP, SN as a within-subject factor) on activation differences. Again, the order of treatment was included to control for possible order effects.

Additional analyses on genotype-treatment interactions

While effects of genotype and treatment were examined on absolute stimulus ratings and brain activation compared to a low-level baseline, we were also interested in potential genotype-treatment interactions on differential responses to the shown stimuli. That is, genotype might moderate the influence of treatment on the *difference* in ratings between neutral and painful/emotional stimuli, and the difference in corresponding brain responses. To increase sensitivity, we restricted our analyses to pre-defined regions of interest (ROI).

To examine these potential effects on the brain level, we computed β-images contrasting the painful or emotional stimuli to the respective neutral stimuli for each experiment, i.e. physical pain > physical neutral, happy faces > neutral faces, sad faces > neutral faces, and social pain > social neutral. For each contrast, we defined a random-effects GLM including treatment as a within-subject factor, resulting in four separate general linear models examining activation differences as a function of treatment. In a next step, we extracted averaged contrast estimates within the ACC, anterior insula, and amygdala to obtain an estimate of global activation of the entire ROIs. This was done separately for the oxytocin and placebo session within each of the four contrasts. The number of risk alleles was coded in a linear fashion (AA<GA<GG) and correlated with contrast estimates after oxytocin and placebo administration, using Spearman's rho as a nonparametric measure. The correlation coefficients for oxytocin and placebo were then tested for significant differences[14]. Significantly different correlations of genotype and brain activation depending on the treatment condition would suggest a significant genotype × treatment interaction.

We used a parallel approach to examine these effects in stimulus ratings. First, we calculated individual differences in ratings for neutral and painful/emotional stimuli separately for the oxytocin and placebo session. Again, the number of risk alleles was coded in a linear fashion (AA<GA<GG) and correlated (Spearman's rho) with rating differences after oxytocin and placebo administration. For each rating difference, the correlation coefficients after oxytocin and placebo administration were then tested for differences[14].

In an exploratory approach, we also used this method to examine potential interactions of symptom severity (ADOS severity score) and treatment. Results of all analyses are reported in Supplementary Tables S1-S4.

SUPPLEMENTARY TABLES

Table S1. Spearman correlations of *OXTR* genotype and rating differences for all experiments

		Oxytocin		Placebo		*z*	*p'*
		rho	*p*	*rho*	*p*		
Physical pain	Pain-Neutral	-.021	.920	-.060	.776	0.20	.844
Basic emotions	Sad-Neutral	-.156	.477	.362	.090	-2.25	.098
	Happy-Neutral	-.194	.376	-.369	.084	1.10	.544
Social pain	Pain-Neutral	-.498	.022	-.019	.934	-1.91	.167

Note. *z*-values and *p*-values in the last three columns derive from asymptotic *z*-tests comparing correlations of mean activation with *OXTR* genotype after oxytocin and placebo administration[14,16]. *p*-values are adjusted for multiple comparisons using the Holm-Bonferroni correction while keeping the significance level of $p < .05$ constant[17]. Data derive from $N = 25$ patients for the physical pain experiment, $N = 23$ for the basic emotions experiment, and $N = 21$ for the social pain experiment.

Table S2. Correlations of ADOS severity scores and rating differences for all experiments

		Oxytocin		Placebo			
		rho	*p*	*rho*	*p*	*z*	*p'*
Physical pain	Pain-Neutral	-.369	.069	.119	.572	-2.60	.038
Basic emotions	Sad-Neutral	.068	.757	-.332	.121	1.71	.260
	Happy-Neutral	.030	.891	.171	.436	-0.85	.796
Social pain	Pain-Neutral	-.345	.126	-.128	.580	-0.83	.796

Note. ADOS = Autism Diagnostic Observational Schedule[18,19]. Higher scores indicate higher symptom severity. *z*-values and corresponding *p*-values derive from asymptotic *z*-tests comparing correlations of mean activation with ADOS sum score after oxytocin and placebo administration[14,16]. *P'*-values are adjusted for multiple comparisons using the Holm-Bonferroni correction while keeping the significance level of $p < .05$ constant[17]. Data derive from $N = 25$ patients for the physical pain experiment, $N = 23$ for the basic emotions experiment, and $N = 21$ for the social pain experiment.

Table S3. Spearman correlations of *OXTR* genotype and mean activation in regions of interest for all experiments

			Oxytocin		Placebo			
			rho	*p*	*rho*	*p*	*z*	*p*
Physical pain	Pain-Neutral	ACC	-.091	.665	-.152	.467	0.22	.413
		left AI	-.013	.952	-.242	.243	0.66	.508
		right AI	-.032	.880	-.150	.474	0.35	.726
		left amygdala	.068	.746	-.141	.503	0.59	.278
		right amygdala	.102	.627	-.259	.212	1.10	.135
Basic emotions	Sad-Neutral	ACC	.089	.687	.305	.157	-.640	.522
		left AI	.001	.996	.195	.372	-.547	.584
		right AI	.054	.806	-.051	.817	.285	.776
		left amygdala	.060	.787	.121	.581	-.214	.830
		right amygdala	.234	.282	.052	.814	.583	.560
	Happy-Neutral	ACC	-.065	.768	-.190	.386	0.44	.662
		left AI	.070	.749	-.008	.973	0.24	.812
		right AI	-.010	.965	.064	.772	-0.21	.835
		left amygdala	.213	.330	.166	.449	0.17	.862
		right amygdala	.048	.829	.223	.305	-0.63	.529
Social pain	Pain-Neutral	ACC	.162	.482	-.006	.980	0.53	.598
		left AI	-.004	.988	.011	.963	-0.04	.965
		right AI	.132	.569	.016	.945	0.36	.721
		left amygdala	.091	.694	.065	.779	0.09	.932
		right amygdala	-.047	.839	.112	.628	-0.57	.572

Note. ACC = anterior cingulate cortex, AI = anterior insula. *z*-values and *p*-values in the last column derive from asymptotic *z*-tests comparing correlations of mean activation with OXTR genotype after oxytocin and placebo administration[103]. *p*-values shown here are not corrected for multiple comparisons. After Holm-Bonferroni correction, all *p*s = 1.000, which represents an upper-bound estimate. Data derived from $n = 25$ patients for the physical pain experiment, $n = 23$ for the basic emotions experiment, and $n = 21$ for the social pain experiment.

Table S4. Spearman correlations of ADOS comparison scores and mean activation in Regions of Interest for all experiments

			Oxytocin		Placebo			
			rho	*p*	*rho*	*p*	*z*	*p'*
Physical pain	Pain-Neutral	ACC	-.337	.100	.237	.255	-2.17	.600
		left AI	-.283	.171	.223	.284	-1.48	1.000
		right AI	-.217	.297	.154	.461	-1.11	1.000
		left amygdala	-.209	.315	.119	.572	-0.93	1.000
		right amygdala	-.102	.627	.121	.565	-0.67	1.000
Basic emotions	Sad-Neutral	ACC	.056	.799	.162	.460	-0.31	1.000
		left AI	.044	.843	.101	.646	-0.16	1.000
		right AI	.080	.718	.259	.232	-0.50	1.000
		left amygdala	.206	.345	.401	.058	-0.62	1.000
		right amygdala	-.001	.996	.262	.227	-0.84	1.000
	Happy-Neutral	ACC	.098	.655	.144	.511	-0.37	1.000
		left AI	.155	.480	.045	.838	0.34	1.000
		right AI	.156	.476	-.162	.462	0.90	1.000
		left amygdala	-.133	.544	.194	.376	-1.20	1.000
		right amygdala	-.038	.863	.229	.294	-0.96	1.000
Social pain	Pain-Neutral	ACC	-.086	.709	-.024	.916	-0.19	1.000
		left AI	-.141	.543	-.304	.180	0.50	1.000
		right AI	-.112	.628	-.181	.431	0.22	1.000
		left amygdala	-.311	.170	-.292	.198	-0.07	1.000
		right amygdala	-.348	.122	-.050	.829	-1.10	1.000

Note. ADOS = Autism Diagnostic Observational Schedule[18,19]. A higher comparison score on the ADOS indicates higher symptom severity. ACC = anterior cingulate cortex, AI = anterior insula. *z*-values and corresponding *p*-values derive from asymptotic *z*-tests comparing correlations of mean activation with ADOS comparison scores after oxytocin and placebo administration[14,16]. *P'*-values are adjusted for multiple comparisons using the Holm-Bonferroni correction while keeping the significance level of p <.05 constant[17]. *P'*-values of 1.000 denote upper bound estimates. Data derive from N = 25 patients for the physical pain experiment, N = 23 for the basic emotions experiment, and N = 21 for the social pain experiment.

Table S5. Brain activation related to empathy for physical pain

Anatomical region	Cyto Area	Side	Cluster size	MNI coordinates x	y	z	*T*	*p* (FWE)
Anterior cingulate cortex		L	532	-8	26	28	6.18	< .001
Superior medial gyrus		L		-6	18	42	6.05	.001
Posterior-medial frontal		R		6	18	48	5.57	.003
Supramarginal gyrus		R	10	64	-20	44	5.40	.006
Inferior frontal gyrus (p. triangularis)		L	30	-44	38	14	5.27	.010
Insula		L	36	-30	20	6	5.22	.012
Precentral gyrus		L	4	-56	6	32	5.16	.015
Supramarginal gyrus	Area PFt	L	16	-62	-22	34	5.06	.021
Middle frontal gyrus		L	8	-44	46	0	5.03	.024
Precentral gyrus		L	1	-58	-36	52	4.84	.045
Precentral gyrus	Area 44	L	1	-48	12	4	4.83	.047

Note. Results of the contrast Physical Pain > Physical Neutral in the whole brain, model accounting for genotype. All statistics are family-wise error (FWE) corrected for whole-brain analyses at the voxel level. The “Cyto Area”-column indicates the cytoarchitectonical area as assigned by the SPM Anatomy toolbox v2.2b[20] if available. Anatomical labels were derived respectively.

Table S6. Brain activation related to faces during basic emotions experiment

Anatomical region	Cyto Area	Side	Cluster size	MNI coordinates x	y	z	*T*	*p* (FWE)
Superior occipital gyrus	hOc1 [V1]	R	18261	20	-98	6	21.09	< .001
Middle occipital gyrus	hOc1 [V1]	L		-12	-98	0	20.22	< .001
Middle occipital gyrus	hOc3d [V3d]	L		-22	-98	12	18.79	< .001
Inferior frontal gyrus (p. opercularis)		R	3585	46	8	28	9.08	< .001
Inferior frontal gyrus (p. triangularis)		R		48	30	26	7.86	< .001
Insula		R		40	22	-6	6.76	< .001
Posterior-medial frontal		L	1458	-2	12	50	9.02	< .001
Midcingulate cortex		R		6	24	38	7.62	< .001
Thalamus	Thal: temporal	R	216	20	-30	0	8.47	< .001
Insula		L	1801	-30	22	4	7.33	< .001
Precentral gyrus		L		-46	8	34	7.27	< .001
Inferior frontal gyrus (p. orbitalis)		L		-36	22	-6	6.64	< .001
Thalamus	Thal: visual	L	290	-20	-30	0	7.12	< .001
Thalamus	Thal: prefrontal	L		-14	-20	10	6.00	< .001
Thalamus	Thal: prefrontal	R	52	10	-10	10	5.23	.007
Middle frontal gyrus		L	37	-42	48	2	5.19	.008
Superior temporal gyrus		R	10	48	-40	14	4.85	.029
Inferior frontal gyrus (p. triangularis)		L	2	-44	36	16	4.75	.042

Note. Results of a conjunction analysis over all facial stimuli (Sad ∩ Happy ∩ Emotional Neutral), model accounting for genotype. All statistics are family-wise error (FWE) corrected for whole-brain analyses at the voxel level. The “Cyto Area”-column indicates the cytoarchitectonical area as assigned by the SPM Anatomy toolbox v2.2b[20] if available. Anatomical labels were derived respectively.

Table S7. Brain activation related to empathy for social pain

Anatomical region	Cyto Area	Side	Cluster size	MNI coordinates x	y	z	*T*	*p* (FWE)
Middle temporal gyrus		L	884	-56	-58	8	8.13	< .001
Middle temporal gyrus		L		-54	-44	8	6.15	< .001
Thalamus	Thal: prefrontal	L	1113	-8	-10	8	6.55	< .001
Caudate nucleus		L		-10	8	4	6.35	< .001
		L		-10	0	8	6.09	< .001
Posterior-medial frontal		L	650	-6	24	54	6.49	< .001
Superior medial gyrus		L		-6	20	42	6.10	< .001
Posterior-medial frontal		L		-6	14	58	5.79	.001
Inferior frontal gyrus (p. opercularis)		L	571	-44	6	24	6.37	< .001
	Area 45	L		-52	18	-4	5.66	.001
Inferior frontal gyrus (p. triangularis)		L		-46	20	6	5.53	.002
Superior medial gyrus		L	133	-6	50	22	6.06	< .001
Temporal pole		R	83	50	8	-22	5.75	.001
Temporal pole		R		46	14	-26	5.30	.006
Middle temporal gyrus		R	204	56	-54	2	5.39	.004
Middle temporal gyrus	Area PGa	R		58	-50	10	5.36	.005
Inferior temporal gyrus	Area FG4	L	42	-42	-44	-14	5.14	.011
Fusiform gyrus	Area FG4	L		-44	-48	-22	4.90	.028
Supramarginal gyrus	Area PF	L	26	-60	-36	36	5.01	.018
Midcingulate cortex		R	9	12	20	38	5.01	.019
Inferior frontal gyrus (p. triangularis)	Area 45	R	12	54	36	4	4.99	.020
Superior temporal gyrus		R	8	48	-24	-4	4.92	.025
Fusiform gyrus	Area FG4	L	4	-44	-58	-18	4.87	.031
Supramarginal gyrus	Area PFm	R	3	54	-44	24	4.86	.031
Middle temporal gyrus		R	3	48	-34	2	4.82	.036

Note. Results of the contrast Social Pain > Social Neutral, model accounting for genotype. All statistics are family-wise error (FWE) corrected for whole-brain analyses at the voxel level. The “Cyto Area”-column indicates the cytoarchitectonical area as assigned by the SPM Anatomy toolbox v2.2b[20] if available. Anatomical labels were derived respectively.

Table S8. Effects of oxytocin and placebo nasal spray on mood and affective states

		Oxytocin				Placebo						
		pre		post		pre		post				
		M	*SD*	*M*	*SD*	*M*	*SD*	*M*	*SD*	*t*	*p'*	*d*
MDBF	good-bad mood	34.28	4.92	35.96	4.02	34.33	5.94	35.08	4.02	0.72	1.000	0.22
	awake-sleepy	31.04	5.48	30.36	6.78	32.42	4.81	29.79	6.18	1.34	.970	0.31
	calm-agitated	33.00	5.22	35.40	4.03	32.54	6.98	34.08	4.90	0.86	1.000	0.25
SAM	valence	6.83	1.81	7.17	1.13	7.08	1.14	7.25	1.19	0.54	1.000	0.15
	arousal	3.75	1.85	3.46	2.11	3.92	2.02	3.46	2.09	0.31	1.000	0.09
	dominance	6.08	1.79	7.00	1.56	6.29	1.92	6.46	2.06	2.34	.168	0.54

Note. MDBF = Multidimensional Mood Questionnaire (Mehrdimensionaler Befindlichkeitsfragebogen)[4]; assessment tool measuring three dimensions of current mood: pleasant-unpleasant, awake-sleepy, and calm-agitated. Higher scores indicate good mood, wakefulness, and calmness, respectively. SAM = Self-Assessment-Manikin[5]; pictorial assessment technique measuring the current state of pleasure, arousal, and dominance. *t*, *p*, and *d*-values derive from paired *t*-tests comparing the change of affect/mood after oxytocin and placebo administration. The change of affect/mood was quantified as the difference in ratings after vs. before substance administration. Positive *t*- and *d*-values indicate higher rating differences after oxytocin compared to placebo. Values in the *p'*-column represent *p*-values adjusted for multiple comparisons using the Holm-Bonferroni correction while keeping the significance level of $p < .05$ constant[17]. *p'*-values of 1.000 denote upper bound estimates. *N* = 24.

Table S9. Mean blood pressure and heart rate before and after substance administration

		Oxytocin								Placebo							
		T0		T1		T2		T3		T0		T1		T2		T3	
Blood pressure (mmHg)	systolic	121.5	(9.2)	118.7	(9.3)	115.0	(9.3)	122.6	(7.1)	118.0	(9.1)	117.9	(9.8)	115.1	(8.7)	121.0	(9.1)
	diastolic	75.8	(7.1)	75.4	(6.3)	72.6	(7.3)	76.2	(6.0)	71.8	(7.3)	73.3	(6.5)	72.9	(7.3)	75.7	(8.6)
Heart rate (bpm)		72.8	(10.8)	69.5	(11.6)	68.3	(12.6)	70.1	(9.2)	71.6	(11.9)	67.5	(11.6)	67.1	(10.9)	66.8	(9.6)

Note. Means and standard deviations (in brackets) for blood pressure and heart rate data. Blood pressure and heart rate were measured four times during study visits. T0 = baseline measure at the beginning of the visit, T1 = immediately after substance administration, T2 = 10-20 minutes after substance administration, prior to MRI scanning, T3 = ~90 minutes after substance administration, immediately after MRI scanning. Effects of treatment and time were examined using two-way repeated-measures ANOVAs. These analyses yielded significant main effects of time on blood pressure [systolic: $F(3,24)= 8.88$, $p < .001$, partial $\eta^2 = .270$; diastolic: $F(3,24)= 2.91$, $p = .040$, partial $\eta^2 = .108$] and heart rate [$F(2.26,24)= 6.76$ (Greenhouse-Geisser corrected for violations of sphericity), $p = .002$, partial $\eta^2 = .220$]. There were no significant main effects of treatment or interaction effects of treatment and time [all *p*s > .162]. $N = 25$.

Table S10: Physical Pain: Comparison of results across different models of stimulus ratings controlling for age, dose, and treatment order.

	Original model		+ age		+ dose		+ order	
	F	η^2_p	*F*	η^2_p	*F*	η^2_p	*F*	η^2_p
Within-subject effects								
Category	901.89***	0.975	22.00***	0.500	460.34***	0.954	1036.23***	0.979
Category*Genotype	0.04	0.002	0.01	<0.001	<0.01	<0.001	0.03	0.002
Treatment	2.31	0.091	1.80	0.076	0.06	0.003	2.11	0.088
Treatment*Genotype	1.17	0.048	0.77	0.034	0.25	0.011	1.76	0.074
Treatment*Category	3.97	0.147	0.27	0.012	0.52	0.023	3.54	0.139
Between-subject effects								
Genotype	1.42	0.058	0.92	0.040	0.60	0.026	1.35	0.058
Covariate	.	.	6.493*	0.228	0.62	0.027	<0.01	<0.001

Note. ***$p < .001$; ** $p < .01$; *$p < .05$. The original model included treatment (oxytocin, placebo) and stimulus category (physical pain, physical neutral) as within-subject factors, and genotype (AA<GA<GG) as a covariate. The additional models were identical to the original model but included participant age, received dose, or treatment order as one additional covariate. "No change in effects" was defined as (a) no change in significance (no new significant effects, no loss of significant effects compared to original model), and (b) no change in effect size (effect size [η^2_p] of significant effects is not altered by more than 50% compared to original model). To be consistent with fMRI analyses, we did not model three-way interactions. $N = 25$.

Table S11: Basic Emotions: Comparison of results across different models of stimulus ratings controlling for age, dose, and treatment order.

	Original model		+ age		+ dose		+ order	
	F	η^2_p	*F*	η^2_p	*F*	η^2_p	*F*	η^2_p
Within-subject effects								
Category	486.80***	0.959	26.77***	0.572	288.08***	0.935	461.31***	0.958
Category*Genotype	1.97	0.086	2.09	0.095	2.53	0.112	1.85	0.085
Treatment	0.70	0.032	0.55	0.027	0.06	0.003	0.44	0.021
Treatment*Genotype	2.44	0.104	2.53	0.112	1.34	0.063	2.30	0.103
Treatment*Category	8.10**	0.278	1.21	0.057	3.97*	0.166	7.87**	0.282
Between-subject effects								
Genotype	0.95	0.043	1.42	0.066	4.75*	0.192	0.88	0.042
Covariate	.	.	2.79	0.123	7.65*	0.277	0.03	0.002

Note. ****p* < .001; ** *p* < .01; **p* < .05. The original model included treatment (oxytocin, placebo) and stimulus category (happy, sad, neutral) as within-subject factors, and genotype (AA<GA<GG) as a covariate. The additional models were identical to the original model but included participant age, received dose, or treatment order as one additional covariate. "No change in effects" was defined as (a) no change in significance (no new significant effects, no loss of significant effects compared to original model), and (b) no change in effect size (effect size [η^2_p] of significant effects is not altered by more than 50% compared to original model). To be consistent with fMRI analyses, we did not model three-way interactions. *N* = 23.

Table S12: Social Pain: Comparison of results across different models of stimulus ratings controlling for age, dose, and treatment order.

	Original model		+ age		+ dose		+ order	
	F	η^2p	*F*	η^2p	*F*	η^2p	*F*	η^2p
Within-subject effects								
Category	1413.25***	0.987	40.03***	0.690	532.47***	0.967	1485.07***	0.988
Category*Genotype	3.72	0.164	3.38	0.158	3.42	0.160	3.42	0.160
Treatment	3.00	0.136	0.34	0.018	0.45	0.024	2.72	0.131
Treatment*Genotype	1.38	0.068	1.17	0.061	0.63	0.034	1.92	0.096
Treatment*Category	0.40	0.020	0.70	0.037	0.59	0.032	0.60	0.032
Between-subject effects								
Genotype	2.51	0.116	2.20	0.109	0.16	0.009	0.56	0.030
Covariate	.	.	1.36	0.070	1.40	0.072	2.64	0.128

Note. ***p < .001; ** p < .01; *p < .05. The original model included treatment (oxytocin, placebo) and stimulus category (social pain, social neutral) as within-subject factors, and genotype (AA<GA<GG) as a covariate. The additional models were identical to the original model but included participant age, received dose, or treatment order as one additional covariate. “No change in effects” was defined as (a) no change in significance (no new significant effects, no loss of significant effects compared to original model), and (b) no change in effect size (effect size [η^2_p] of significant effects is not altered by more than 50% compared to original model). To be consistent with fMRI analyses, we did not model three-way interactions. N = 21.

SUPPLEMENTARY FIGURES

Figure S1. CONSORT Flow Diagram of patient recruitment and study flow.

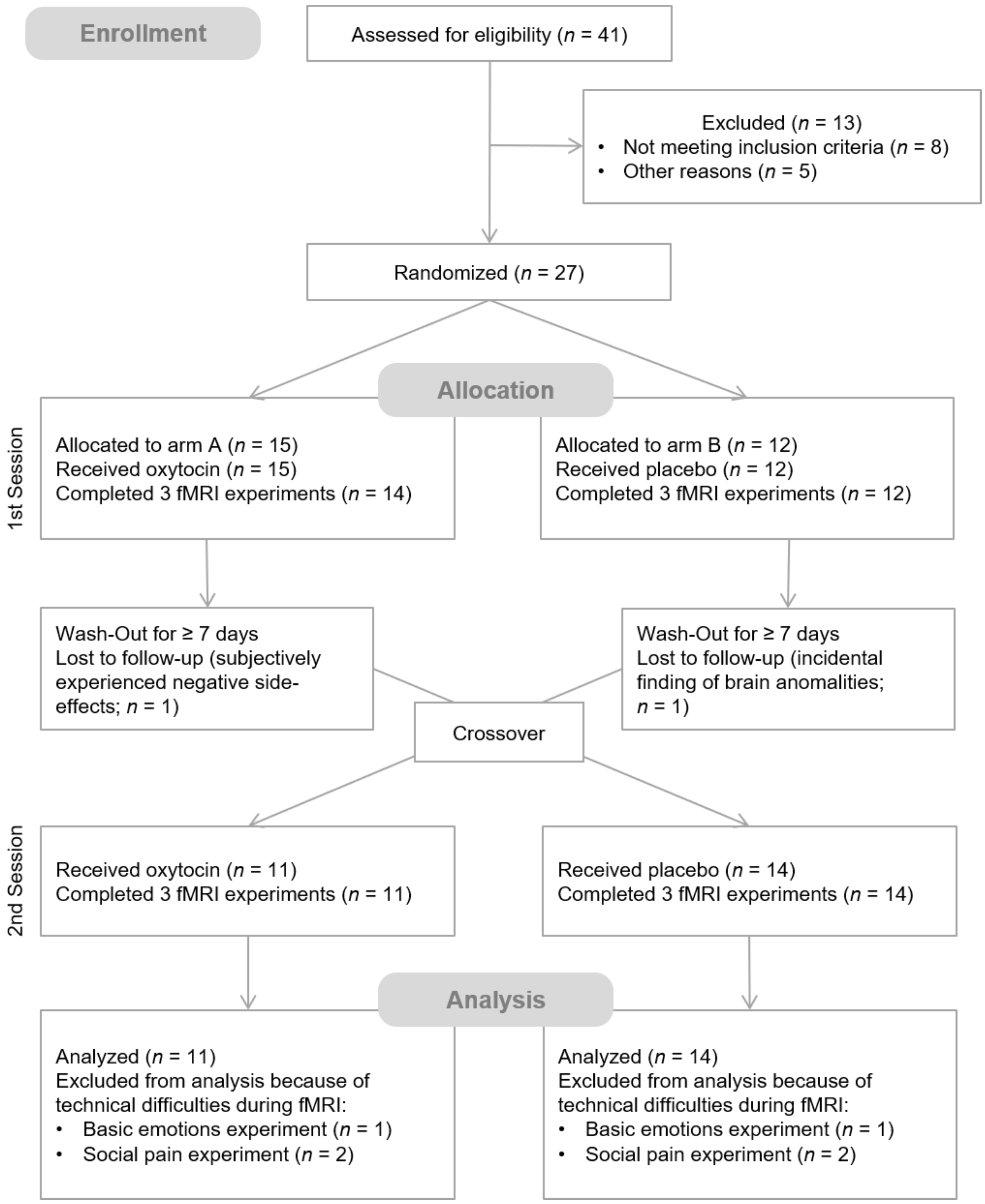

Figure S2. Physical Pain: Comparison of significant effects across different models of brain activation controlling for age, dose, and treatment order.

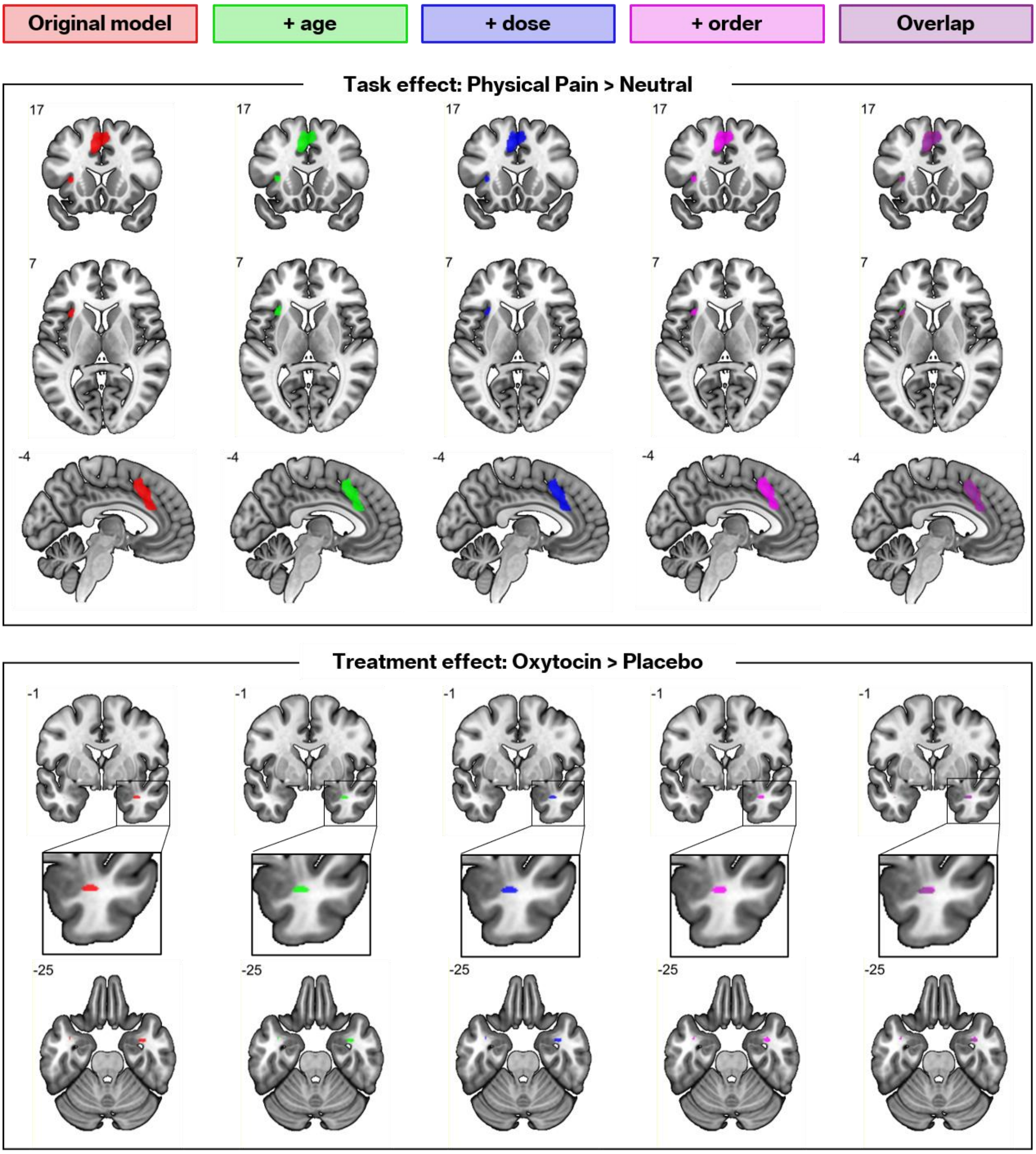

In our main analyses, we used a general linear model that included treatment and stimulus category as within-subject factors, and genotype (AA<GA<GG) as a covariate ("original model", red). Physically painful situations, compared to neutral control situations, induced activation of anterior cingulate cortex and left anterior insula (top). Further, oxytocin enhanced activation within the right amygdala and left amygdala/hippocampus (bottom). Controlling for age (green), dose (blue), and order of treatment (pink) did not change the magnitude of these task and treatment effects, as can be seen from the large overlap in the statistical maps ("overlap", purple). "No change in effects" was defined as (a) no change in significance (no new significant clusters with $k > 5$, no loss of significant clusters with $k > 5$ compared to

original model); (b) no change in location and cluster size (significant clusters have roughly same size [±10% of total voxels] and location, as indicated by visual inspection). Results are displayed at voxel-level *p*(FWE) < .05 across the whole brain. Brain images were created with MRIcroGL (https://www.nitrc.org/projects/mricrogl/).

Figure S3. Basic Emotions: Comparison of significant effects across different models of brain activation controlling for age, dose, and treatment order.

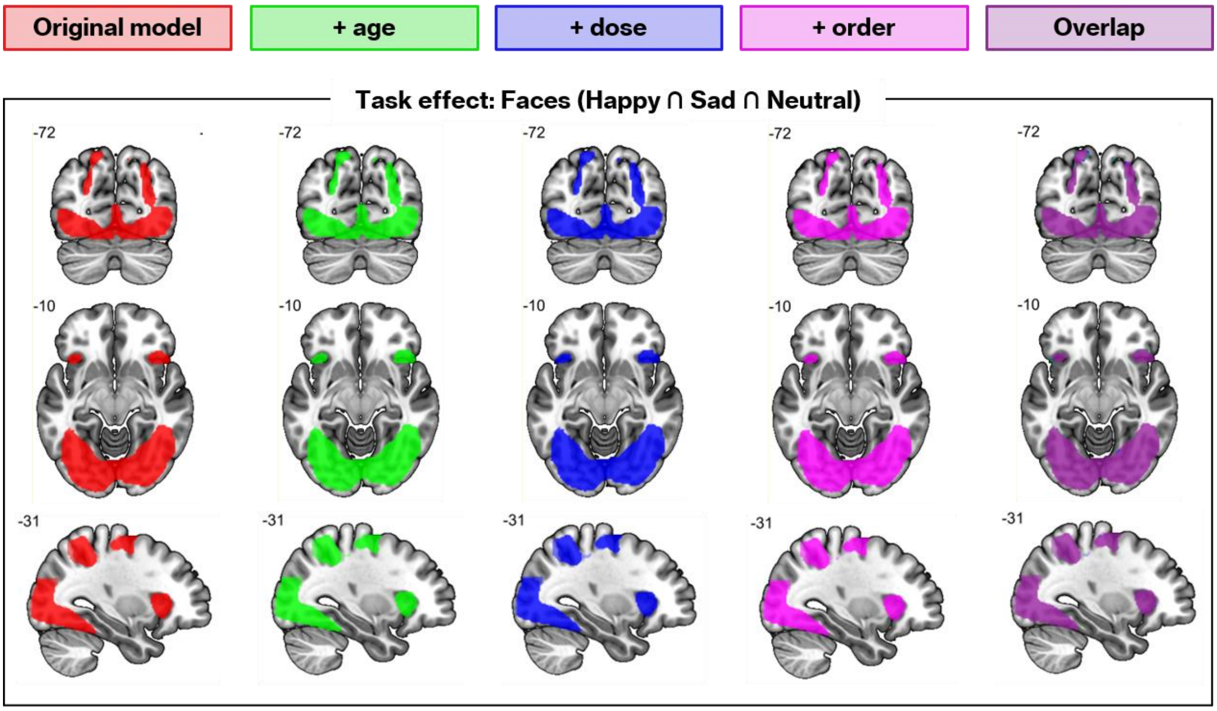

In our main analyses, we used a general linear model that included treatment (oxytocin, placebo) and stimulus category as within-subject factors, and genotype (AA<GA<GG) as a covariate (“original model”, red). Faces induced widespread activity within occipital regions, including the fusiform and occipital face area, as well as inferior frontal regions, anterior insula, and medial cingulate cortex. There were no statistically significant effects of oxytocin treatment. Controlling for age (green), dose (blue), and order of treatment (pink) did not change the magnitude of the task effect, as can be seen from the large overlap in the statistical maps (“overlap”, purple). “No change in effects” was defined as (a) no change in significance (no new significant clusters with k > 5, no loss of significant clusters with k > 5 compared to original model); (b) no change in location and cluster size (significant clusters have roughly same size [±10% of total voxels] and location, as indicated by visual inspection). Results are displayed at voxel-level *p*(FWE) < .05 across the whole brain. Brain images were created with MRIcroGL (https://www.nitrc.org/projects/mricrogl/).

Figure S4. Social Pain: Comparison of significant effects across different models of brain activation controlling for age, dose, and treatment order.

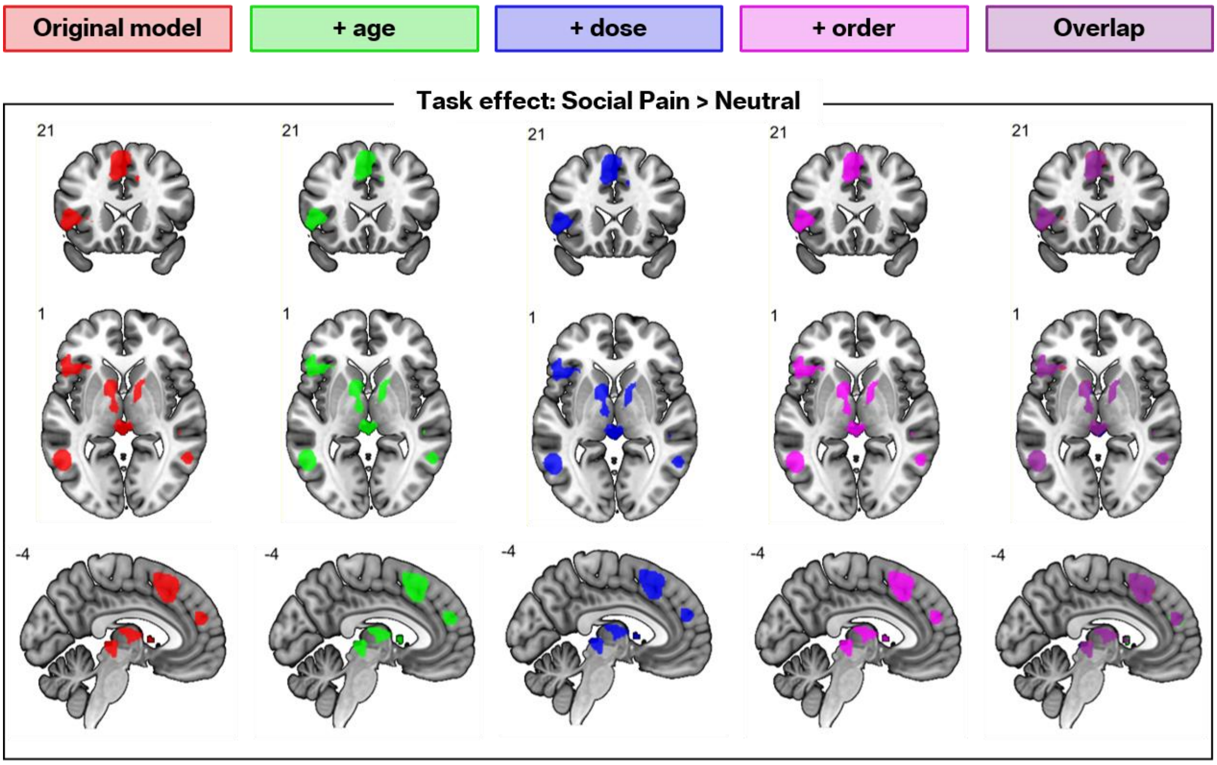

In our main analyses, we used a general linear model that included treatment (oxytocin, placebo) and stimulus category as within-subject factors, and genotype (AA<GA<GG) as a covariate ("original model", red). Socially painful situations, compared to neutral situations, elicited significant activation in posterior temporal regions, posterior medial frontal gyrus, thalamus, inferior frontal gyrus, and supramarginal gyrus. There were no statistically significant effects of oxytocin treatment. Controlling for age (green), dose (blue), and order of treatment (pink) did not change the magnitude of the task effect, as can be seen from the large overlap in the statistical maps ("overlap", purple). "No change in effects" was defined as (a) no change in significance (no new significant clusters with k > 5, no loss of significant clusters with k > 5 compared to original model); (b) no change in location and cluster size (significant clusters have roughly same size [±10% of total voxels] and location, as indicated by visual inspection). Results are displayed at voxel-level *p*(FWE) < .05 across the whole brain. Brain images were created with MRIcroGL (https://www.nitrc.org/projects/mricrogl/).

References

1. Faul, F., Erdfelder, E., Lang, A.-G. & Buchner, A. G*Power: A flexible statistical power analysis program for the social, behavioral, and biomedical sciences. *Behav. Res. Methods* **39**, 175–191 (2007).
2. Bakermans-Kranenburg, M. J. & van I Jzendoorn, M. H. Sniffing around oxytocin: review and meta-analyses of trials in healthy and clinical groups with implications for pharmacotherapy. *Transl. Psychiatry* **3**, (2013).
3. Wechsler, D. *Wechsler intelligence scale for children–Fourth Edition (WISC-IV)*. (The Psychological Corporation, 2003).
4. Steyer, R., Schwenkmezger, P., Notz, P. & Eid, M. *Der Mehrdimensionale Befindlichkeitsfragebogen (MDBF). Handanweisung.* (Hogrefe, 1997).
5. Bradley, M. M. & Lang, P. J. Measuring emotion: The self-assessment manikin and the semantic differential. *J. Behav. Ther. Exp. Psychiatry* **25**, 49–59 (1994).
6. Paulus, F. M. *et al.* Laugh or cringe? Common and distinct processes of reward-based schadenfreude and empathy-based fremdscham. *Neuropsychologia* **116**, 52–60 (2018).
7. Krach, S. *et al.* Your flaws are my pain: Linking empathy to vicarious embarrassment. *PLoS One* **6**, e18675 (2011).
8. Paulus, F. M., Müller-Pinzler, L., Jansen, A., Gazzola, V. & Krach, S. Mentalizing and the role of the posterior superior temporal sulcus in sharing others' embarrassment. *Cereb. Cortex* **25**, 2065–2075 (2015).
9. Müller-Pinzler, L., Paulus, F. M., Stemmler, G. & Krach, S. Increased autonomic activation in vicarious embarrassment. *Int. J. Psychophysiol.* **86**, 74–82 (2012).
10. Krach, S. *et al.* Evidence from pupillometry and fMRI indicates reduced neural response during vicarious social pain but not physical pain in autism. *Hum. Brain Mapp.* **36**, 4730–4744 (2015).
11. Technische Universität Chemnitz & Einhäuser-Treyer, W. Visually-induced pain empathy repository. (2016). Available at: https://www.tu-chemnitz.de/physik/PHKP/viper.html. (Accessed: 22nd July 2019)
12. Lundqvist, D., Flykt, A. & Öhman, A. The Karolinska directed emotional faces (KDEF). *CD ROM from Dep. Clin. Neurosci. Psychol. Sect. Karolinska Institutet* **91**, 630 (1998).
13. Paulus, F. M., Kamp-Becker, I. & Krach, S. Demands in reflecting about another's motives and intentions modulate vicarious embarrassment in autism spectrum disorders. *Res. Dev. Disabil.* **34**, 1312–1321 (2013).
14. Lee, I. A. & Preacher, K. J. Calculation for the test of the difference between two dependent correlations with one variable in common. (2013). Available at: http://quantpsy.org.
15. Hus, V. & Lord, C. The Autism Diagnostic Observation Schedule, Module 4: Revised Algorithm and Standardized Severity Scores. *J. Autism Dev. Disord.* **44**, 1996–2012 (2014).
16. Steiger, J. H. Tests for comparing elements of a correlation matrix. *Psychological Bulletin* **87**, 245–251 (1980).
17. Holm, S. A simple sequentially rejective multiple test procedure. *Scand. J. Stat.* **6**, 65–70 (1979).
18. Lord, C. *et al.* Autism Diagnostic Observation Schedule (ADOS). *J. Autism Dev. Disord.* (2000). doi:10.1007/BF02211841.
19. Rühl, D., Bölte, S., Feineis-Matthews, S. & Poustka, F. *Diagnostische Beobachtungsskala für Autistische Störungen (ADOS).* (Huber, 2004).
20. Eickhoff, S. B. *et al.* A new SPM toolbox for combining probabilistic cytoarchitectonic maps and functional imaging data. *Neuroimage* **25**, 1325–1335 (2005).
